# Factors Influencing the Transition From Physiological Hyperopia to Myopia in Children: Protocol for a Prospective Nested Case-Control Study

**DOI:** 10.2196/84888

**Published:** 2026-03-24

**Authors:** Huaying Xu, Jianing Pu, Shimeng Bian, Xuejing Mi, Zhen Zhou, Wei Chen, Yonghong Jiao

**Affiliations:** 1 Department of Ophthalmology, Beijing Tongren Eye Center, Capital Medical University, Beijing Tongren Hospital, 2 West Ring South Road, Beijing Economic and Technological Development Zone, Beijing, 100730, China; 2 Department of Ophthalmology, Maternal and Child Health Hospital of Haidian District, Beijing, China; 3 School of Biomedical Engineering, Capital Medical University, Beijing, China

**Keywords:** physiological hyperopia, myopia, children, risk factor, nested case-control study

## Abstract

**Background:**

The early onset of myopia in children has become a critical public health issue that requires urgent attention. Notably, high myopia-related retinal diseases have emerged as the leading cause of irreversible blindness in adults in certain regions of China. Physiological hyperopia, as a protective factor and one of the strongest predictors of myopia development, plays a key role in delaying the progression of early-onset myopia and reducing the risk of high myopia in adulthood. However, the dynamic changes, critical turning points, and factors contributing to the rapid regression of physiological hyperopia during childhood remain unclear.

**Objective:**

This study aims to explore the key influencing factors for children’s physiological hyperopia fading and myopia onset, as well as the protective mechanisms of physiological hyperopia against myopia.

**Methods:**

This was a prospective nested case-control study. Our research team previously established a prospective cohort of 2109 preschool children, aged 3 to 6 years, through cluster sampling in 22 kindergartens in Haidian District, Beijing, and completed a 3-year follow-up. Building on this cohort, this study adopts a prospective nested case-control design. We will continue to follow up this cohort with a 3-year longitudinal study, tracking children aged 6 to 9 years. During the 3-year follow-up period, participants will undergo annual eye examinations and complete questionnaires regarding their living habits and environment. The primary outcome is incident myopia, while secondary outcomes include the prevalence of myopia and the changes in various ocular biological parameters. The study received ethics approval on April 7, 2024, from the Ethics Committee of Beijing Tongren Eye Center, Beijing Tongren Hospital (TREC2024-KY034) and was registered on July 5, 2024.

**Results:**

Participant recruitment began on July 10, 2024, and is expected to be completed by December 31, 2026. As of February 2026, recruitment is ongoing, and the final results are expected by December 2026.

**Conclusions:**

This prospective nested casecontrol study investigates the dynamic changes and regression patterns of physiological hyperopia in school-aged children and evaluates the associations between ocular biometric parameters and the onset of myopia. The findings are expected to support standardized myopia screening, inform clinical interventions, and provide evidence-based guidance for government policies on myopia prevention and control in young children.

## Introduction

Myopia is a major global public health problem and is projected to affect approximately 50% of the world’s population by 2050 [1]. It is the most common ocular condition in many Asian countries, with prevalence rates substantially higher in regions such as China, Singapore, Hong Kong, and Taiwan than in Western countries [2,3]. A 2025 nationwide analysis of more than 5 million Chinese children and adolescents reported a persistently high prevalence of myopia, with a steep age-related increase and projections indicating a continued rise in high myopia, highlighting the long-term public health burden in China [4].

Myopia adversely affects the physical and psychological well-being of children and adolescents and may lead to serious ocular complications, including retinal detachment, macular degeneration, glaucoma, and cataract [5-7]. In some regions of China, high myopia-associated retinopathy has become the leading cause of irreversible blindness in adults [8], resulting in a marked reduction in quality of life. Myopia is an irreversible ocular condition, and treatment options are limited to controlling its progression and preventing complications after onset [9]. Therefore, early prevention and intervention of myopia in children in China are urgent public health priorities.

Physiologically, newborns typically present with hyperopia, with an average refractive error of approximately +2.50 to +3.00 diopters (D). With increasing age, hyperopia gradually decreases and progresses toward emmetropia (−0.50 to +0.50 D) [5]. However, environmental and behavioral changes in modern society—including increased exposure to artificial lighting, widespread use of electronic devices, reduced outdoor activity, and intensified early educational demands—have substantially altered this developmental process [10-13]. In some children, these factors lead to an early onset of emmetropia, which often persists only briefly before progressing to myopia. This premyopic hyperopic state, referred to as physiological hyperopia, may serve as an important predictor of early myopia onset [14]. From an epidemiological perspective, reducing disease incidence is central to effective prevention. In childhood myopia, this strategy translates into preventing myopia among children who are not yet affected. Accordingly, the trajectory of physiological hyperopia during early childhood represents a critical target for myopia prevention. However, the mechanisms underlying physiological hyperopia changes, the timing of critical transition points, and the factors driving rapid physiological hyperopia regression and subsequent myopia development remain poorly understood.

In China, childhood myopia screening and prevention are guided by national public health policies. The Myopia Prevention and Control Guidelines (2024 edition) issued by the National Health Commission recommend regular vision screening in kindergarten and primary school settings and emphasize early identification of children at risk for myopia. These national recommendations are implemented locally through school-based vision screening programs in cities such as Beijing. However, evidence on the longitudinal trajectory of physiological hyperopia and its transition to myopia during the critical preschool to school-age period remains limited.

Therefore, this study conducted a nested case-control investigation among school-aged children in the Haidian District of Beijing to establish a cohort of children with physiological hyperopia. The baseline values of refractive error, visual acuity, axial length (AL), anterior chamber depth (ACD), corneal radius (CR), and other ocular biometric parameters were recorded following cycloplegia. Through longitudinal follow-up, this study aims to identify key factors influencing physiological hyperopia regression and myopia onset, as well as to explore the protective role of physiological hyperopia in myopia development. The findings are expected to provide evidence to inform population-level strategies for early myopia prevention and control.

## Methods

### Objective

This study aims to explore the key influencing factors for children’s physiological hyperopia fading and myopia onset, as well as the protective mechanisms of physiological hyperopia against myopia.

### Study Design

This study is a prospective observational study based on an established cohort, with planned nested case-control analyses embedded within the longitudinal follow-up framework. All participants are followed annually using standardized ophthalmic examinations and questionnaires. Incident myopia cases identified during follow-up are used for the primary inferential analyses through risk-set matched nested case-control sampling, while cohort-level and cross-sectional analyses are conducted to describe longitudinal refractive and biometric changes. The primary inferential analyses are conducted using risk-set matched nested case-control sampling focused on incident myopia. The study period spans from January 1, 2024, to December 31, 2026, and the protocol is registered at ClinicalTrials.gov (NCT06498947).

Our research team previously established a prospective cohort of 2109 preschool children, aged 3 to 6 years, through cluster sampling in 22 kindergartens in Haidian District, Beijing, and completed a 3-year follow-up. Building on this established cohort, this study continues prospective follow-up as children transition into school age (6‐9 years), with annual ophthalmic examinations and questionnaire-based assessments.

Although all participants are followed longitudinally, the primary scientific objective of this study is not to model population-average trajectories alone, but to identify factors associated with the transition from physiological hyperopia to incident myopia during this critical developmental window. For this purpose, a prospective nested case-control design was selected as the main inferential strategy within the cohort framework.

The nested case-control study is an innovative research method that combines elements of traditional case-control and cohort studies, and its validity has been demonstrated in multiple large-scale studies [15-17]. Nested case-control sampling enables efficient and focused comparison between children who develop myopia and those who remain nonmyopic at the same follow-up time point, thereby preserving the temporal sequence between exposure and outcome while minimizing selection bias. In addition, key exposures of interest—such as baseline hyperopic reserve and the rate of physiological hyperopia regression—are age dependent and time varying. Risk-set matching within the cohort facilitates appropriate control for age and follow-up time and supports robust multivariable modeling of detailed exposure profiles.

Case-control sampling is performed only when new cases of myopia are identified during follow-up. Each incident myopia case is matched in a 1:1 ratio with a control selected from cohort members who remain at risk at the same time point. Cross-sectional analyses conducted at each follow-up visit are intended solely for descriptive and exploratory purposes and do not constitute part of the primary inferential analyses.

### Study Setting and Participants

This study focuses on school-aged children in Haidian District, Beijing, and includes a 3-year prospective follow-up. As the educational and technological hub of northwest Beijing, Haidian District has a well-established education management system, facilitating research coordination. Furthermore, the high level of parental compliance with myopia prevention measures in the region ensures the successful implementation of long-term follow-up.

**Figure 1.**
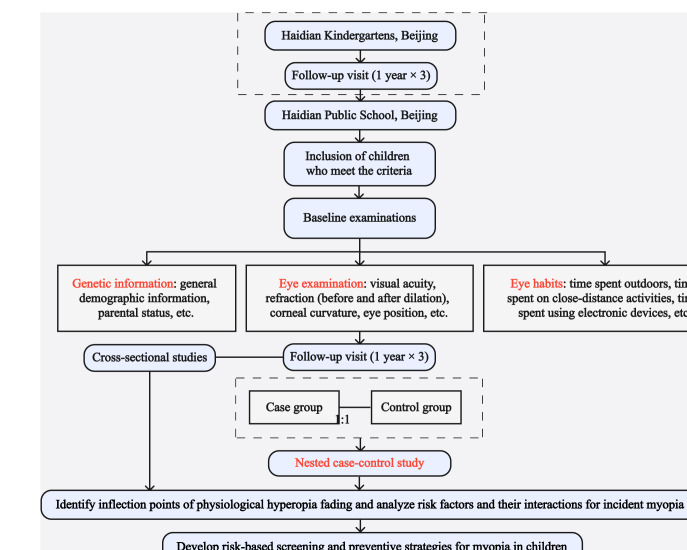
Flowchart of the study.

Infants typically exhibit a hyperopia of +3.0 to +4.0 D, which transitions into a period of rapid development before the age of 3 years, followed by a slower developmental phase. By approximately 12 years, the process of emmetropization is gradually completed. On the basis of this developmental pattern, this study focuses on school-aged children as the target population. For this study, children aged 6 to 9 years who met the eligibility criteria were invited to continue annual follow-up examinations for an additional 3 years. Figure 1 illustrates the overall participant flow, including cohort enrollment, eligibility assessment, annual follow-up visits, and identification of incident myopia cases for nested case-control analyses. Textbox 1 lists the inclusion and exclusion criteria.

Textbox 1. **Inclusion and exclusion criteria.**

**Inclusion criteria**

- Children aged 6 to 9 years, regardless of sex
- Ability to cooperate during examinations
- Parental consent obtained through signed informed consent

**Exclusion criteria**

- Children with a history of drug allergies
- Children with heart disease, cranial trauma, epilepsy, Down syndrome, or glaucoma
- Children with a history of myopia prevention interventions (eg, atropine or peripheral defocus corrective lenses)

### Sample Size Calculation

The sample size was estimated based on the planned analyses of incident myopia over the 3-year follow-up in the prospective cohort, with prespecified 1:1 matching in the nested case-control component. Published epidemiological studies indicate that myopia prevalence is relatively low in preschool-aged children but increases rapidly after school entry. For example, Lan et al [18] reported that the prevalence of myopia among 3-year-old children was <2.5%, whereas He et al [19] reported a prevalence of 5.9% among 6-year-old children and an average prevalence of approximately 36% among primary school students in Guangzhou.

On the basis of these data and the anticipated number of incident myopia cases during follow-up, the minimum required cohort size was estimated to be 915 participants to support multivariable analyses and the prespecified nested case-control comparisons. Allowing for an anticipated 10% loss to follow-up, the final target sample size was increased to 1006 participants. This cohort size is expected to generate a sufficient number of incident myopia cases during follow-up to allow for 1:1 risk-set matching and adjustment for key confounders.

### Outcome Measures

#### Primary Outcome

The primary outcome is incident myopia, defined as the development of myopia during follow-up among participants who were nonmyopic at baseline. Myopia is defined as a spherical equivalent refraction (sphere +1/2 cylinder) of ≤−0.50 D in either eye under cycloplegia [20].

#### Secondary Outcomes

The secondary outcomes assessed in this study include the following:

- Prevalence of myopia at baseline and each annual follow-up visit [4]
- Annual change in AL [21]
- Annual change in ACD [22]
- Annual change in CR [23]
- Annual change in the AL to CR ratio [23]

All outcomes are assessed annually throughout the 3-year follow-up period using standardized protocols.

### Examinations

Parameters measured included cycloplegic refraction, recording of refractive changes, visual acuity, postdilated refraction, AL, ACD, CR, eye position, fundus, height, and weight.

All the abovementioned metrics were collected at each follow-up visit, and data were collected annually for a specified period.

### Questionnaires

#### General Information Questionnaire

Participants’ general information was collected, including name, sex, gestational age at birth, mode of delivery, birth weight, whether the parents were myopic, the degree of myopia, and the health of the mother during pregnancy.

#### Myopia-Related Genetic and Environmental Factors Questionnaire

These data were collected using Children’s Myopia-Related Factors Questionnaire, which parents completed by scanning the QR code; the questionnaire includes past history, family history, close-distance activities, outdoor activity time, reading habits, and education.

### Data Collection and Follow-Up

This study does not involve any intervention. All procedures are observational and consist of standardized ophthalmic examinations and questionnaire-based assessments conducted at baseline and at each annual follow-up visit. The following biological parameters were measured annually in children in the cohort: visual acuity, cycloplegic refraction, AL, ACD, CR, ocular position, fundus, height, weight, and other parameters.

First, visual acuity was assessed using the Early Treatment Diabetic Retinopathy Study visual acuity chart at a distance of 4 m, with the 1.0 line at eye level. Visual acuity was assessed monocularly, with one eye covered without applying pressure while the other eye was tested. Measurements were performed in a right-to-left sequence. The number of correctly identified letters was determined by reading optotypes from top to bottom.

Second, cycloplegic refraction was performed using 1% cyclopentolate hydrochloride. Initially, 1 drop of 0.4% oxybuprocaine hydrochloride (as topical anesthetic) was instilled. Following a 3-minute interval, 1% cyclopentolate hydrochloride eye drops were administered at 5- to 10-minute intervals for 3 consecutive doses. Pupillary diameter and light reflex were assessed 30 minutes after the final instillation. Complete cycloplegia was defined as pupillary dilation ≥6.0 mm and absence of pupillary light reflex (grade 0 on the standardized light reaction scale). Participants meeting these criteria underwent automated refraction using a calibrated autorefractor (Nidek ARK-1), with triplicate measurements obtained under mesopic conditions (3 cd/m²).

Third, ocular biometric parameters, including AL, CR, and ACD, are measured using an optical interferometric AL meter (AL-Scan, Nidek Co, Ltd). The AL to CR ratio is calculated as AL-to-CR=AL/mean CR. The device automatically obtained multiple measurements and reported averaged values.

Fourth, eye position was assessed using masking-demasking and alternating masking methods to screen for strabismus. The direction of ocular movement was assessed to determine whether the participant had strabismus or occult strabismus.

Data collection was completed annually within the specified time frame, with a total follow-up period of 3 years. To ensure patient adherence and maintain a high long-term follow-up rate, the following strategies were implemented: (1) collaborating institutions had extensive screening experience and a dedicated professional examination team; (2) a personalized refractive profile was established for each child in the cohort, along with a feedback mechanism for results; (3) volunteer certificates were awarded to children participating in the cohort study to enhance their engagement; and (4) an additional clinical coordinator was appointed to conduct detailed telephone communication with families before each annual follow-up, aiming to reduce lost follow-up rates. These measures also support the feasibility and acceptability of long-term follow-up in school-aged children and their families.

### Quality Control Measures and Data Management

The research team established standard operating procedures for the study and organized training programs, with records maintained in the project management files. To ensure that study personnel are adequately qualified to perform specific clinical research tasks, all involved staff must complete the following training before initiation of the project: (1) study protocol; (2) ophthalmic examination standard operating procedures, including protocols for cycloplegic refraction, visual acuity assessment, AL measurement, ACD, and other biometric parameter measurements; and (3) electronic database use. Training related to the project will be ongoing throughout the study, with the principal investigator and project coordinator adding content as needed.

The purpose of this study is to propose data management methods based on the Smart Vision Information OPERA-MC Adolescent Vision Screening and Monitoring Management System. Through an electronic questionnaire, we collected basic information about patients, their families, and their environment. Automated refraction (Nidek ARK-1) and noncontact tonometry (Canon TX-20P; Canon Medical Systems) were digitally interfaced via the standardized HL7 protocol through networked medical device ports. Digital fundus photography images (Topcon TRC-50DX; Topcon Healthcare, Inc) were automatically transmitted and archived in digital imaging and communications in medicine format using picture archiving and communication system integration. Examination results were transmitted electronically for direct collection and storage without manual data entry. This process improved data accuracy and timeliness while reducing the likelihood of missing data. We will check the accuracy and completeness of the database storage results by sampling the original data and comparing them with the stored data. The collected data will be recorded in case report forms and uploaded to the electronic data capture system for centralized management.

### Statistical Analysis Plan

**Figure 2.**
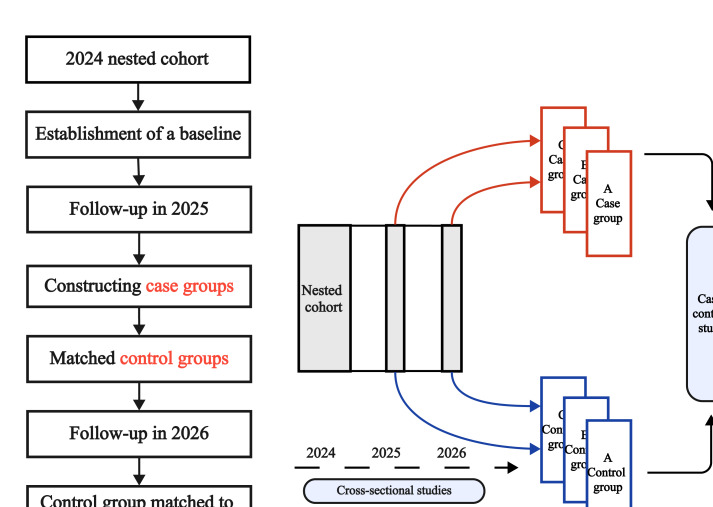
The basic statistical design plan of the study.

In this project, the basic statistical design plan was a prospective nested case-control study, as shown in Figure 2. Statistical analyses will be conducted according to prespecified research questions and outcomes. Analyses will include descriptive analyses, primary inferential analyses for incident myopia, and secondary analyses for longitudinal changes in ocular biometric parameters.

Descriptive analyses will summarize participants’ baseline characteristics and annual follow-up measurements, including refractive status and ocular biometric parameters. Continuous variables will be reported as means (SDs) or medians (IQRs), as appropriate, and categorical variables will be summarized as frequencies and proportions. The distribution of continuous variables will be assessed using graphical methods (eg, histograms and Q-Q plots) and the Shapiro-Wilk test.

The primary inferential analysis will evaluate the association between physiological hyperopia and incident myopia. A nested case-control approach will be applied, in which children who develop myopia during follow-up will be identified as cases and matched in a 1:1 ratio with controls selected from cohort members who remain nonmyopic at the same follow-up time point. Conditional logistic regression models will be used to estimate odds ratios and 95% CIs, adjusting for potential confounders, including age, sex, parental myopia, time spent in outdoor activities, and time spent on near-work activities.

Secondary analyses will examine longitudinal changes in ocular biometric parameters, including AL, ACD, CR, and the AL to CR ratio. These analyses will be performed using linear mixed-effects models to account for within-subject repeated measurements over time. Participants will be grouped according to baseline levels of physiological hyperopia, and sensitivity analyses will be conducted accordingly.

Sensitivity analyses will be performed by stratifying participants according to baseline physiological hyperopia levels (eg, age-specific categories) and by evaluating alternative analytic-eye definitions (right eye only vs both eyes using appropriate correlation structures).

All statistical analyses will be performed using SPSS (version 26.0; IBM Corp), RStudio (version 1.4; Posit PBC), and MATLAB (version 2021a; MathWorks Inc). A 2-sided *P*<.05 will be considered statistically significant.

### Ethical Considerations

Throughout the study, all procedures were in accordance with the Declaration of Helsinki.

The study received ethics approval on April 7, 2024, from the Ethics Committee of Beijing Tongren Eye Center, Beijing Tongren Hospital, (TREC2024-KY034), and was registered on July 5, 2024. In addition to explaining the purpose and methods of the study, the researchers also explained possible side effects and precautions online before each test. Informed consent was obtained before using cyclopentolate eye drops. No financial or material compensation was provided to participants for their participation in this study.

Medical records and datasets will be maintained at Beijing Tongren Hospital. No personal information will be revealed in any public results report. Data will be examined for authenticity before being given to the study statistician. Data will be kept strictly confidential and evaluated by study staff and authorized personnel. All data files will be password protected and stored on a secure server for a minimum of 10 years and disposed of if they are no longer required. If changes are made to the project, they will be published in ClinicalTrials.gov.

## Results

Participant recruitment began on July 10, 2024, and is expected to be completed by December 31, 2026. As of February 2026, recruitment is ongoing. Data analysis will commence after the completion of data collection, and the final results are expected to be published by December 31, 2026.

## Discussion

### Anticipated Findings

This study has several key advantages. First, it incorporates the regression trajectory of physiological hyperopia into the research framework, using a prospective cohort design to track its association with the onset and progression of myopia. This approach systematically reveals the dynamic changes in the emmetropization process in children, providing vital scientific evidence for the development of myopia prevention strategies for young children. Second, the study uses internationally standardized cycloplegic refraction methods to accurately measure physiological hyperopia in children, along with multiple ocular biometric parameters. The data obtained help fill a gap in the understanding of basic ocular development parameters in young children. Third, this study integrates longitudinal cohort follow-up with prospectively planned nested case-control analyses, allowing focused investigation of risk factors associated with the transition from physiological hyperopia to incident myopia during a critical developmental period. This approach provides a solid methodological foundation for establishing causal relationships in changes in physiological hyperopia.

Several interventions are currently used to address childhood myopia, including behavioral strategies (such as increasing outdoor activity and optimizing near-work habits), optical interventions (eg, orthokeratology and spectacle or contact lenses designed to induce peripheral myopic defocus), and pharmacological approaches, most commonly low-dose atropine eye drops [24-27]. These interventions have demonstrated efficacy in slowing myopia progression after onset. However, they are typically implemented once myopia has already developed and therefore do not directly address the identification of children at high risk before disease onset. This study complements existing intervention strategies by focusing on early refractive development and the role of physiological hyperopia as a potential marker for early myopia risk stratification.

Physiological hyperopia has a protective effect on the development of myopia in children. In a 13-year longitudinal study of kindergarten and school children aged 3 to 17 years, conducted between 1984 and 1996, Matsumura et al [28] found a significant increase in the incidence of myopia among children aged 7 years or older. Myopia development was most prominent in the −1 and −2 D groups, while the change in refractive status was minimal in the +1 D group. This suggests that a certain degree of physiological hyperopia may protect against the onset of myopia. Li et al [29] conducted a 5-year annual follow-up study involving 2835 first-grade students from Anyang City to identify potential risk factors for myopia. They found that the 5-year incidence of myopia was lowest in children with a baseline spherical equivalent refraction greater than +2.00 D (4.4%), and it increased to nearly 92% in children with a baseline spherical equivalent refraction between 0.00 and −0.50 D. These findings highlight the importance of monitoring hyperopic refractive changes in children before they enter elementary school, especially in the first grade.

Previous evidence suggests that the lower the level of physiological hyperopia, the earlier the onset of myopia. Population-based evidence from the Refractive Error Study in Children conducted by Morgan et al [3] in Australia suggests that mild hyperopia (+0.5 to +2.0 D), the typical end point of refractive development in children, may still pose a risk of progressing to myopia if emmetropia develops during childhood. Zadnik et al [30] in the United States conducted a 21-year longitudinal study of 4512 ethnically diverse nonmyopic schoolchildren in grades 1 through 8 to assess risk factors for myopia onset. They found that lower baseline hyperopia was associated with a higher risk of developing myopia, suggesting that future myopia prevention trials should target children with low levels of hyperopia, that is, physiological hyperopia deficiency. Therefore, during the critical period of refractive transition from preschool to school age—before myopia actually develops—further research is needed to identify children at high risk of myopia based on relevant risk factors and to determine effective strategies to preserve physiological hyperopia to delay or prevent the onset of myopia.

### Limitations

This study has several limitations that should be considered when interpreting the findings. First, although the cohort is relatively large and well characterized, participants were recruited from a single district in Beijing. Therefore, the findings may not be fully generalizable to children in other regions with different socioeconomic, educational, or environmental contexts. Second, this study is observational in nature. While the prospective design and nested case-control analyses strengthen temporal inference, causal relationships between physiological hyperopia–related exposures and myopia onset cannot be definitively established, and residual confounding may persist despite multivariable adjustment. Third, physiological hyperopia and related environmental exposures were assessed at discrete annual time points. More frequent measurements might better capture short-term fluctuations in refractive development and behavioral factors. In addition, some exposure information was collected via parent-reported questionnaires, which may be subject to recall or reporting bias. Finally, although standardized cycloplegic refraction and ocular biometry were used, analytic decisions such as eye selection and categorization of physiological hyperopia may influence effect estimates. However, sensitivity analyses were conducted to assess the robustness of the findings to these analytic choices.

### Conclusions

This prospective nested case-control study investigates the dynamic changes and regression patterns of physiological hyperopia in school-aged children and evaluates the associations between ocular biometric parameters and the onset of myopia. The findings are expected to support standardized myopia screening, inform clinical interventions, and provide evidence-based guidance for government policies on myopia prevention and control in young children.

## Supplementary material

10.2196/84888 Checklist 1 STROBE checklist.

## Abbreviations

ACD: anterior chamber depth
AL: axial length
CR: corneal radius
D: diopter
SER: spherical equivalent refractive
